# Reno-protective effect and mechanism study of Huang Lian Jie Du Decoction on lupus nephritis MRL/lpr mice

**DOI:** 10.1186/s12906-016-1433-1

**Published:** 2016-11-09

**Authors:** Xiaoli Nie, Rong Deng, Lei Xiang, Pingping Jiang, Qi Xue

**Affiliations:** 1 School of Traditional Chinese Medicine, Southern Medical University, Guangzhou, 510515 People’s Republic of China; 2 Department of Traditional Chinese Medicine, Nanfang Hospital, Southern Medical University, Guangzhou, 510515 People’s Republic of China; 3 Department of General Surgery, Nanfang Hospital, Southern Medical University, Guangzhou, 510515 People’s Republic of China

**Keywords:** Huang Lian Jie Du Decoction, Lupus nephritis, MRL/lpr mice, Immune function, *p*-STAT 3

## Abstract

**Background:**

Huang Lian Jie Du Decoction (HLJDD), a very famous traditional Chinese medicinal prescription, has been used for heat dissipation and detoxification in China. This study was aimed to evaluate the reno-protective effects of HLJDD against lupus nephritis (LN) in vivo in MRL/lpr mice.

**Methods:**

Animals were administered orally every day for eight consecutive weeks except the mice of normal group and model group. Organ indexes, serum interleukin-6 (IL-6), interleukin-10 (IL-10), interferon-gamma (IFN-γ) and the anti-double stranded DNA (anti-dsDNA) antibody were tested, respectively. Creatinine (Cr), blood urea nitrogen (BUN) and urine protein were measured for renal function evaluation. The expression of phosphorylated signal transducer and activator of transcription 3 (*p*-STAT 3) in kidney tissue was observed by western blot (WB) and immunohistochemical (IHC) method. Meanwhile, histopathological changes in the renal were studied by hematoxylin-eosin (H&E) staining.

**Results:**

The mice of HLJDD-treated group exhibited a significant reduced mortality (*p* < 0.05), serum anti-dsDNA level (*p* < 0.05) and renal immune complex deposition (*p* < 0.05), compared with the untreated MRL/lpr mice. In addition, HLJDD treatment remarkably reduced the levels of BUN, Cr, proteinuria (*p* < 0.01) and the levels of inflammatory cytokines such as IL-6, IL-10 and IFN-γ (*p* < 0.01). Moreover, HLJDD significantly suppressed the phosphorylations of STAT 3 (*p* < 0.05) and the renal pathological changes.

**Conclusions:**

The study implied that HLJDD may be a potential agent for the therapy of LN, and the down-regulated *p*-STAT 3 expression suggesting that it may be one of the LN therapy targets for HLJDD.

## Background

Systemic lupus erythematosus (SLE) is a chronic multisystem autoimmune disease with the features of antinuclear antibody in serum and pathological changes in multiple organs, such as arthritis, nephritis and rash [1, 2]. The symptoms of SLE are mostly displayed in females with the ages of 15–50, and affects more than 1 million individuals each year [1, 3]. Immune complex deposition, recruitment of neutrophils and complement activation is the main reason for the pathogenesis of SLE [1, 4].

Lupus nephritis (LN), one of the higher frequency complications in SLE, is characterized by glomerulonephritis and tubule-interstitial inflammation combined with the depositing of immune complexes in the tissue of renal [5–8]. The involvement of LN, especially the type of proliferative glomerulonephritis significantly decreased the LN patients life expectancy and their survival [9]. Recently, the prognosis of SLE patients with nephropathy has been markedly improved by the therapy of immunosuppressive. However, the definite strategies for LN treatment remain undefined, and there is still many of patients progress to the end-stage renal disease [8, 10, 11]. Thus, developing an inexpensive natural agents that possesses the protective effects for LN patients is further a higher challenge.

Traditional Chinese medicine (TCM) has been widely used in the treatment of many complex and refractory diseases for thousands of years. Huang Lian Jie Du Decoction (HLJDD), named *Hwangryun-Hae-Dok* Decotion in Japan, is a very recognized traditional Chinese medicinal prescription, mainly used to heat dissipation and detoxification in China for 2000 years. This classical formulation was first described in details in a famous treatise “Wai Tai Mi Yao” by Wang Tao in the Tang Dynasty, and which has been officially listed in Chinese Pharmacopoeia (2005 edition) in the clinical practice of TCM [12]. The decoction consists of four famous traditional Chinese medicinal herbs, namely Huanglian (*Coptis chinensis* Franch, Ranunculaceae) (HL), Huangqin (*Scutellaria baicalensis* Georgi, Labiatae) (HQ), Huangbai (*Phellodendron amurense* Rupr, Rutaceae) (HB) and Zhizi (*Gardenia jasminoides* Ellis, Rubiaceae) (ZZ) in a weight ratio of 3:2:2:3 [13]. In terms of TCM theory, HL used as the major component aimed to balance the body disharmony, HQ acted as the minister component to assist the therapeutic action of the principal medicinal components. The adjuvant and messenger constituent mainly used as the guider to adjust the formulation to the targeted organs or to eliminate the body disharmony caused by other medicines [14]. In HLJDD, HB is classified as adjuvant component and ZZ played both adjuvant and messenger components. The combined effect of HB and ZZ is purposed to directly resolving heat toxin in TCM theory [15].

Currently, HLJDD has attracted lots of attentions in China and other Asia countries because of its potential therapeutic effect in many diseases. Modern pharmacological works displayed that HLJDD possesses widely effects, including the treatment of inflammation [16, 17], gastrointestinal disorders [18], diabetes [17], vasodilation [19], acute liver injury [20], Alzheimer disease [21, 22], hypertension [23], multiple myeloma [24] and other cardiovascular diseases [25, 26]. Until now, some research had reported the content determinations of the main bioactive components contained in HLJDD, in order to understand and elucidate its pharmacological effects and the relevant mechanisms [27, 28]. While, berberine, baicalin, baicalein, wogonoside, wogonin and geniposide have been identified, and been considered as the main bioactive components in HLJDD [27, 29]. In those identified components, berberine and baicalin have been reported to possess anti-inflammatory effects and have been used to treat many diseases [30]. Ni et al. reported that the berberine exhibited a strong renoprotective effects in in vivo diabetic nephropathy model [31]. Baicalin, a flavonoid glycoside isolated from *Scutellaria baicalensis*, showed many biological activities, such as anti-inflammatory [32], immune system modulatory activities [33] and especially great for the protective effect on lead-induced renal oxidative damage in mice [34]. Furthermore, the other main components of HLJDD are mainly exhibited strong anti-inflammation, immune modulatory and protective kidney effect [35–38]. In addition, HLJDD exhibited the impressive anti-inflammatory effect in mice through multi-target and multi-channel actions, due to its multiple constituents (e.g. geniposide, baicalin, berberine, palmatine etc.) in its decoction [17]. And the previous published papers showed that HLJDD exerted beneficial effects on the inflammation model of lipopolysaccharide-induced RAW264.7 cells in vitro [17]. In addition, our preliminary work displayed that HLJDD could reduce the incidence of acute kidney injury, protect the kidneys and regulating organs recovery in in vivo animal model.

With that in mind, our study was played to investigate the potential protective effects by HLJDD with reduces renal pathology and suppresses inflammation reaction in LN MRL/lpr mice. To the best of our knowledge, there is no any previous researches on the reno-protective effects of HLJDD for LN treatment in vivo. This study also aimed to explore the underlying protective mechanisms of HLJDD.

## Methods

### Materials and reagents

The specific mouse enzyme-linked immunosorbent assay (ELISA) kits of IL-6 (KMC0061) and IL-10 (KMC0101) were obtained from Life Technologies, USA. IFN-γ (SEA049Mu) commercial detection kit was purchased from Cloud-Clone corp., USA. Serum BUN (C013-2) and Cr (C011-2) were purchased from Nanjing Jiancheng Bioengineering Institute, Jiangsu, China. BCA protein assay kit was obtained from Zhongshan Institute of Biotechnology, Beijing, China. Carboxymethyl cellulose sodium salt (CMC-Na, C4888) was purchased from Sigma-Aldrich, USA. All other reagents used in these experiments were purchased from Sigma-Aldrich unless otherwise noted.

Prednisone acetate tablet (5 mg prednisone acetate per tablet) was provided by Beijing Union Pharmaceutical Company.

### Herbal informations and preparation methods of HLJDD

The four crude herbal materials of HLJDD, Rhizoma *Coptidis* (HL), Radix *Scutellariae Baicalensis* (HQ), Cortex *Phellodendri Amurensis* (HB) and Fructus *Gardeniae* (ZZ) were purchased from E-fong Pharmaceutical Company (Foshan, China) and authenticated by professor Xiaoli Nie (School of Traditional Chinese Medicine & Department of Traditional Chinese Medicine, Nanfang Hospital, Southern Medical University Guangzhou, China) and Qi Xue (Department of General Surgery, Nanfang Hospital, Southern Medical University, Guangzhou, China). The voucher specimens, deposited at the School of Traditional Chinese Medicine, Southern Medical University, were 20150101-HL, 20150102-HQ, 20150103-HB and 20150104-ZZ for Rhizoma Coptidis, Radix Scutellariae, Cortex Phellodendri and Fructus Gardeniae, respectively.

Briefly, to prepare the HLJDD, four dried crude herbs were ground into powders, mixed in a weight ratio of 3:2:2:3 (HL:HQ:HB:ZZ, reaching a total weight of 1000 g) and extracted twice with boiling water (1:10 and then 1:8, w/v) for 1.5 h. The aqueous extract were combined, concentrated and vacuumized to dryness to afford 285.50 g HLJDD extract (yield: 28.55 %) [13], and then stored in refrigerator at 4 °C. Suspension solution of the extract was made in 0.25 % CMC-Na solution before intragastric administration.

### Animals

Ten weeks old female MRL/lpr mice (23.5 ± 2.0 g) and sex- and age-matched WT C57BL/6 mice (21.5 ± 1.0 g) were purchased from the Model Animal Research Center of Southern Medical University (Guangzhou, China). Mice were fed under specific pathogen-free conditions at 24 ± 1 °C and 50 ± 5 % relative humidity with a 12 h light/dark cycle and with free access to standard water and food adlibitum. All the animals were housed for 1 week under the controlled conditions before experiments. All procedures involving mice in this manuscript were approved by the Committee on the Ethics of Animal Experiments of Southern Medical University (SMU, Protocol Number: 2015063) and were carried out in accordance with the National Institute of Health guidelines (National Research Council of USA, 1996).

### Experimental design

After 1 week acclimatization, twelve WT C57BL/6 mice were used as group A: vehical group (10 ml/kg, 0.9 % saline). And 48 MRL/lpr mice were randomly divided into 4 groups (*n* = 12), including group B: model group (10 ml/kg, 0.9 % saline), group C: HLJDD group (5.4 g/kg, weight ratio between crude drug and rat), group D: HLJDD + prednisone group (5.4 g/kg HLJDD extract +12.5 mg/kg prednisone) and group E: prednisone group (12.5 mg/kg), each experimental group consisted of 12 mice. In addition, the dose of the drug used in the experimental mice was converted by the conversion coefficients table for the dose per kilogram of animal and human body weight [39]. The above groups continuous administration of 8 weeks by gavage with the same volumes (10 ml/kg/day).

All mice were sacrificed by cervical dislocation on the day after the last intragastric administration. The amount of 24 h proteinuria were assessed at weeks of 0, 4 and 8 respectively. Blood and renal tissue samples were collected for further examinations. Serum was obtained by the centrifuge at 3000 rpm for 10 min under 4 °C, and stored at −20 °C before use. Renal tissues were obtained and fixed in 4 % neutral-buffered formalin and embedded in paraffin for histopathological and IHC analysis. Additional renal samples were frozen in liquid nitrogen and stored at −80 °C for biochemical assays. The experimental protocols used in the present study were approved by the Animal Ethical Committee of Southern Medical University.

### Measurement of survival rate and animal general information

The survival rate and the general animal information including animal body weight, skin fur condition, the appearances and behaviors of the animal in each group were recorded at every week during the experiment time.

### Measurement of renal function

Blood serum BUN and Cr levels were measured at 8 weeks using the commercial kit (Nanjing Jiancheng Bioengineering Institute, Jiangsu, China) in an Olympus AU 600 Autoanalyzer, Japan. The observation absorbance of BUN and Cr were read at 510 nm and the content was calculated as mg/dL.

### Measurement of the urinary protein

Urine samples were obtained using metabolic cages in 0, 4 and 8 weeks at 24 h during the whole experiment period before sacrifice respectively. The collected urine samples were centrifuged at 3000 rpm, 5 min to remove all the particulates, then the supernatant was collected and frozen at −20 °C before use. Urinary protein at 24 h was measured by BCA protein assay kit (Zhongshan Institute of Biotechnology, Beijing, China).

### Measurement of inflammatory cytokines in circumference blood

Serum IL-6, IL-10 and IFN-*γ* were detected by using commercial ELISA kits based on the manufacturer’s instructions. Optical density was measured with an ELISA plate scanner at 450 nm (BioTek Elx × 808, BioTek, USA).

### Measurement of serum anti-dsDNA antibody and complement C3 levels

The levels of anti-dsDNA antibodies in serum was determined by ELISA [40], according to the manufacturer’s protocol. Briefly, 5 μg/mL calf thymus dsDNA (Sigma-Aldrich, USA) first pre-coat into 96-well plates, and then add the serum into 96-well plates, the absorbance was measured at 450 nm. Mouse anti-dsDNA monoclone antibody (Chemicon International, USA) was utilized to prepare a reference standard curve, and the anti-dsDNA concentrations were quantified by the standard curve. Normal mouse IgG was considered as negative control. The complement C3 in serum were determined using appropriate commercial ELISA kits (eBioscience, USA) following the manufacturer’s protocols. The absorbance of each sample was read at 450 nm with a microplate spectrophotometer (BioTek Elx × 808, BioTek, USA).

### Histopathological observation

For microscopic examination, mice renal specimens were fixed with 4 % formaldehyde in 0.01 mol/L phosphate buffer (pH 7.2) and embedding in paraffin, cut into 5 μm thick sections, then stained with H&E and imaged under a light microscope for routine histopathological examination. The examination of renal pathology was performed in a blinded fashion by a pathologist.

### Immunohistochemistry analysis

For IHC analysis, formalin-fixed and paraffin-embedded renal sections (5 μm) were mounted on glass slides, then deparaffinized, incubated in 3 % H_2_O_2_, 10 min to quench endogenous peroxidase activity. Then use normal goat serum to block 20 min, and incubated with monoclonal mouse *p*-STAT 3 antibody (Cell Signaling Technology, 1:100 dilution) overnight at 4 °C. After that, then use the second antibody horseradish peroxidase-conjugated goat anti-mouse antibody (Abmart, 1:500 dilution) to incubate 30 min at 37 °C. The biding sites of the antibody could be visualized after incubation with DAB at room temperature (RT) for 10 min. Images were obtained at original magnification of 400× (Olympus BX-51 Microscope, Japan). The cells of renal tissue containing yellow granulation in the endochylema or nucleus were regarded as positive staining. The positive cell numbers were counted with Q500IW image analysis system (BX-51 Olympus, Japan) and Image-Pro Plus v 6.0 software (Media Cyberne tics Inc., Bethesda, MD, USA).

### Western blot analysis

For WB analysis, aliquot the total renal homogenate from each animals were diluted in lysis buffer, which contained the component of 150 mM NaCl, 50 mM Tris–HCl, pH 7.4, 0.1 % SDS, 1 % Triton X-100, 0.1 mM EGTA, 2 mM EDTA, 5 mM NaF, 1 mM Na_3_VO_4_, 5 mM Na_2_PO_4_ and 1 × proteinase inhibitor cocktail (Roche, USA), to a final protein concentration of 2 mg/mL. Equal protein content (30 μg) were separated by SDS-PAGE and transferred to PVDF membrane (Millipore, USA). Membranes were incubated with primary antibody anti-*p*-STAT 3 (1:100) at 4 °C overnight, after RT blocking with 5 % non-fat milk in TBST solution (TBS + 0.1 % Tween-20, pH 7.4) for 2 h. Then, washed the membranes 3 times and incubated with HRP-conjugated secondary antibody at RT for 2 h. Anti-*β*-actin antibody (SantaCruz, USA) was used as internal controls. The bands were visualized after an enhanced chemi-luminescence system (ECL) (Thermo, USA) incubation and semi-quantitated with Image J software (Bio-Rad, USA). The data are expressed as fold changes, normalize to the internal control.

### Statistical analysis

All data were expressed as mean ± S.D. (standard deviation) and all statistical comparisons were analyzed by means of a one-way ANOVA test followed by Dunett’s *t*-test with SPSS 20.0 software. *p* < 0.01 was considered as statistically significant.

## Results

### General observation and mortality of the experimental mice

Hair loss and skin lesions with scab formation are very common in MRL/lpr mice [41]. In the experiment, the abnormal skin manifestation of MRL/lpr mice first mainly displayed as red spots in head, neck, ears, tail and back, and then became larger patches in two weeks, finally changed to scab and fell off. Robust hair loss and the severe damage of the ear skin and the tail were observed in the model group. The group of HLJDD were significantly reduced the skin lesions in MRL/lpr mice with no red patches or spots, no skin fester and no apparent hair loss. These preliminary results suggested that HLJDD had a therapeutic effect in MRL/lpr mice.

**Fig. 1:**
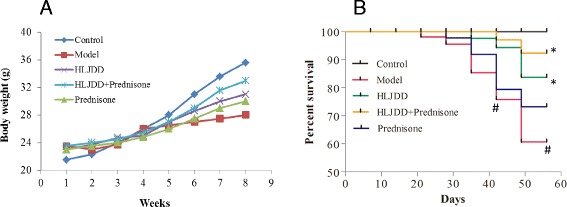
**a** The body weight of the experimental MRL/lpr mice. There is no significant difference in all experimental groups. **b** The survival rate of the experimental MRL/lpr mice Values are presented as mean ± SD. ^#^ *p* < 0.05 versus control group, *p < 0.05 versus model group

Less activity was observed in the model group after 6 weeks, while all group mice behaved normally during 0–5 weeks. There were no significantly differences in body weights among the groups of mice (Fig. 1a), although the body weights of the model and drug-treatment groups were lower than that of the control group by the end of the study.

The effect of HLJDD on the mortality of MRL/lpr mice was investigated. As expected, mortality was observed in all groups except the control group. A significant survival benefit was observed in HLJDD- and HLJDD+ prednisone-treated MRL/lpr mice as compared with the mice in the model group, and the group of HLJDD combined prednisone exhibited the lowest death rate (Fig. 1b). These results indicate that HLJDD significantly increased the survival rate of MRL/lpr mice.

### The effect of HLJDD on spleen coefficient

**Fig. 2:**
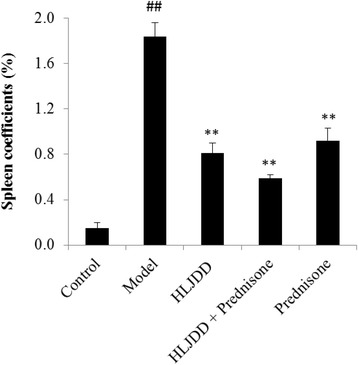
Spleen coefficient (organ weight/body weight × 100 %) of the experimental MRL/lpr mice. Values are presented as mean ± SD. ^##^ *p* < 0.01 versus control group, **p < 0.01 versus model group

In MRL/lpr mice, the spleen weight could increase to about 10 times larger than normal mice, which is one of the most important indexes of SLE progression [42–44]. In the present study, spleen coefficients (organ weight/body weight × 100 %) was significantly enlarged in the model mice (*p* < 0.01, Fig. 2), compared to the normal control mice. While HLJDD-treated group significantly reduced the spleen coefficients, especially in HLJDD combined prednisone group, compared to model group (*p* < 0.01, Fig. 2).

### HLJDD treatment reduced 24 h proteinuria

**Fig. 3:**
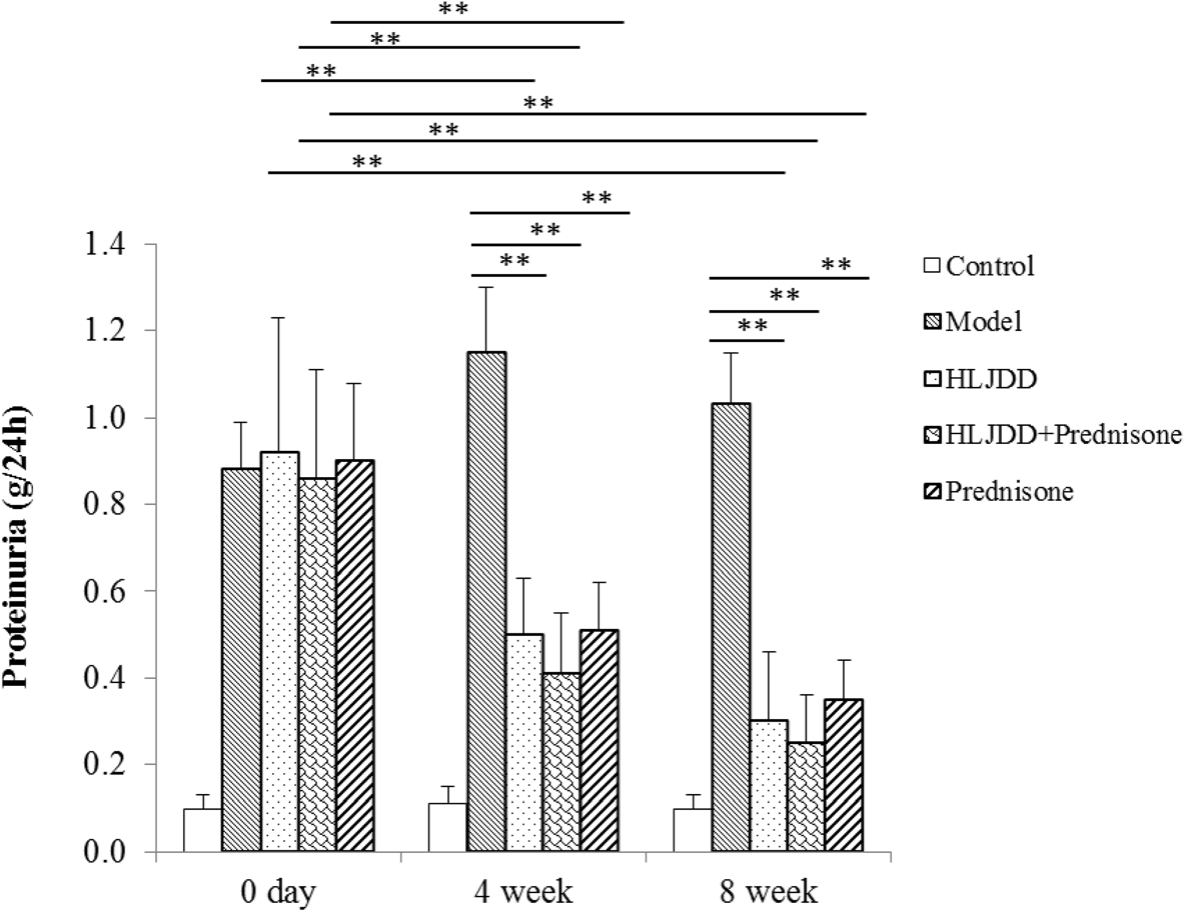
HLJDD reduced 24 h proteinuria in serum of MRL/lpr mice. Twenty-four hour urinary protein was detected by Coomassie Brilliant Blue test at weeks 0, 4 and 8. Values are presented as mean ± SD. ** indicates *p* < 0.01

Proteinuria is a major symptom to indicate the development of renal disease in MRL/lpr mice. In order to investigate the protective effect of HLJDD on the progression of renal disease, Urinary proteins in 24 h were detected every 4 weeks during HLJDD-treated period (from week 0 to 8). As presented in Fig. 3, the amount of 24 h proteinuria exhibited a progressive rise in the model group, compared with C57BL/6 normal group (Fig. 3). While the content of 24 h proteinuria was dramatically decreased in HLJDD-treated groups at week 4, compared with the model group (*p* < 0.01, Fig. 3). At week 8, mice treated with HLJDD had markedly less 24 h proteinuria than the model group mice (*p* < 0.01, Fig. 3). Obviously, the content of 24 h proteinuria in HLJDD-treated mice fell from 0.92 ± 0.25 g/24 h (week 0) to 0.30 ± 0.11 g/24 h (week 8) (*p* < 0.01, Fig. 3), while in HLJDD combined prednisone group, the content of 24 h proteinuria was 0.86 ± 0.18 g/24 h at week 0, and the number declined significantly to 0.41 ± 0.11 g/24 h by week 4 and continued to declined to 0.25 ± 0.09 g/24 h by week 8 (*p* < 0.01, Fig. 3). Taken together, HLJDD demonstrated a significant proteinuria reduction in the progression of SLE in MRL/lpr mice with time-dependence.

### The effect of HLJDD in renal function in MRL/lpr mice

The effect of HLJDD on renal function in MRL/lpr mice was investigated. The level of serum BUN and Cr were markedly increased in model group, compared to the control groups (*p* < 0.01, Table 1). However, there was a notable reduction in renal function in HLJDD-treated MRL/lpr mice (*p* < 0.01, Table 1), especially in the HLJDD combined prednisone group (*p* < 0.01, Table 1). While prednisone-treated group also showed a significate decrease, compared to the model group (*p* < 0.01, Table 1). All the data demonstrate that HLJDD improved the renal function in MRL/lpr mice.

**Table 1:** Effect of HLJDD in renal function in MRL/lpr mice

Groups	BUN (mg/dL)	Cr (mg/dL)
Control	9.93 ± 1.02	9.14 ± 0.88
Model	20.66 ± 2.00^##^	18.22 ± 1.49^##^
HLJDD	12.18 ± 1.11**	11.81 ± 0.92**
HLJDD + Prednisone	10.56 ± 0.85**	10.02 ± 0.67**
Prednisone	13.75 ± 1.30**	12.39 ± 1.03**

Values represent the mean ± S.D. The one-way ANOVA was performed on the raw data

^##^Significant difference at *p* < 0.01 levels compared with the control group

**Significant difference at *p* < 0.01 levels compared with the model group

### The effect of HLJDD on the serum inflammatory cytokines

Considering the important roles of inflammatory cytokines in LN progress [45], the expression levels of serum IL-6, IL-10 and IFN-γ were determined by ELISA. As expected, the expression of serum IL-6, IL-10 and IFN-γ were all significantly increased in the model group, compared to the control group (*p* < 0.01, Table 2). However, compared to the model group, HLJDD-treated group was significantly reduced the expression levels of IL-6, IL-10 and IFN-γ, respectively (*p* < 0.01, Table 2). As similarly, there was also showed a significant difference in prednisone-treated group (*p* < 0.01, Table 2). Furthermore, HLJDD combined prednisone group enhanced the effect of HLJDD on LN, showed a remarkable decrease in these three inflammatory cytokines, compared to the model group (*p* < 0.01, Table 2).

**Table 2:** Effect of HLJDD on the serum inflammatory cytokines

Groups	IL-6 (pg/mL)	IL-10 (pg/mL)	IFN-γ (pg/mL)
Control	18.96 ± 1.54	180.13 ± 6.33	0.65 ± 0.02
Model	78.55 ± 4.01^##^	642.57 ± 10.22^##^	18.23 ± 1.50^##^
HLJDD	34.75 ± 1.11**	211.33 ± 5.34**	1.28 ± 0.17**
HLJDD + Prednisone	34.36 ± 1.85**	201.08 ± 4.73**	1.12 ± 0.09**
Prednisone	35.11 ± 2.30**	222.45 ± 5.15**	1.44 ± 0.04**

Values represent the mean ± S.D. The one-way ANOVA was performed on the raw data

^##^Significant difference at *p* < 0.01 levels compared with the control group

**Significant difference at *p* < 0.01 levels compared with the model group

### The effect of HLJDD on the levels of anti-dsDNA antibody and complement C3 in the serum

**Fig. 4:**
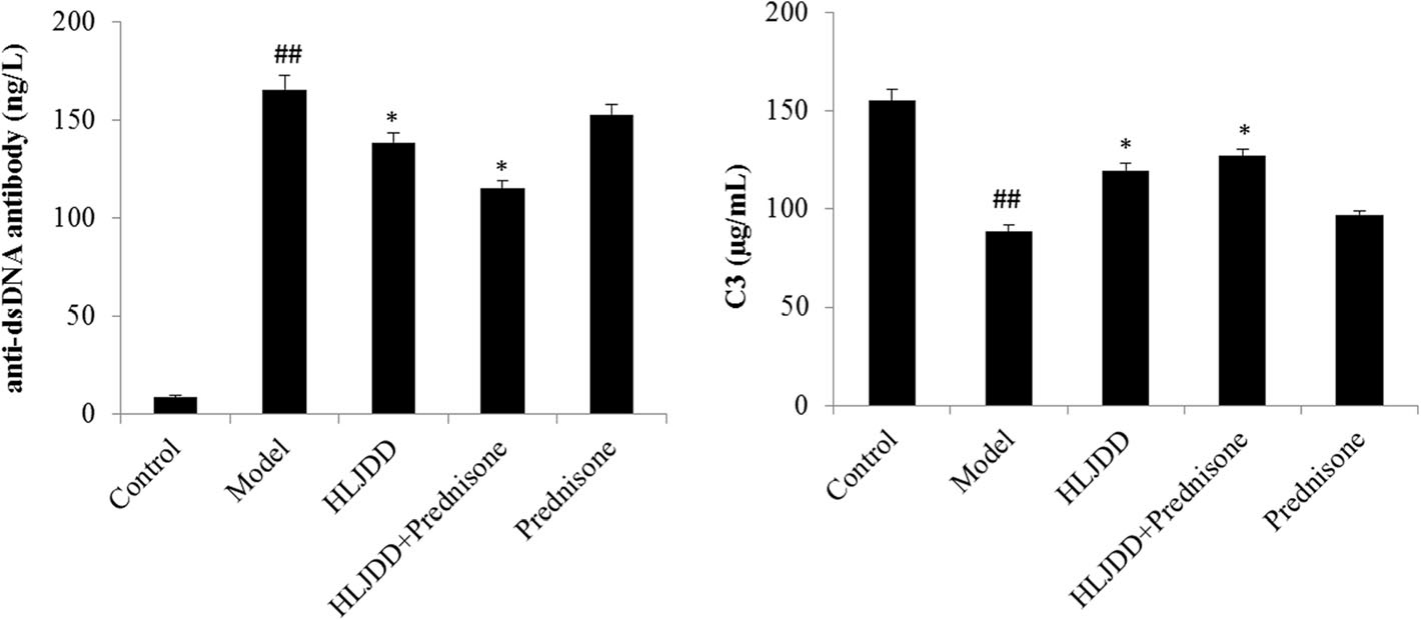
Effect of HLJDD on the levels of anti-dsDNA antibody and complement C3 in the serum. Values are presented as mean ± SD. ^##^ *p* < 0.01 versus control group, **p < 0.01 versus model group

Autoantibody production is a sensitive clinical measurement for SLE, which is closely related to LN activity [46, 47], thus, the anti-dsDNA antibodies and the deposition of C3 concentrations in serum were determined. As shown in Fig. 4, anti-dsDNA antibody in serum was not detected in C57BL/6 control group, which means that there was no LN changes, whereas the anti-dsDNA antibody levels in model group mice exhibited an obvious evidence of LN (*p* < 0.01, Fig. 4). HLJDD decreased the production of anti-dsDNA, especially in HLJDD combined prednisone group (*p* < 0.05, Fig. 4). Furthermore, compared to the control group, the levels of complement C3 in serum in model mice were significantly decreased (*p* < 0.01, Fig. 4). While the low levels of complement C3 were recovered in HLJDD-treated mice, compared to model group (*p* < 0.05, Fig. 4).

### Histopathological studies

**Fig. 5:**
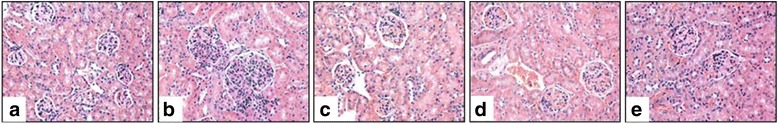
The H&E (×400) of the kidney sections in MRL/lpr mice. **a** Control group; **b** Model group; **c** HLJDD group; **d** HLJDD + Prednisone group; **e** Prednisone group

Furthermore, to assess inflammation in MRL/lpr mice, H&E staining was used to detect the pathologic changes of the tissue of kidneys at the end of the study. Mice in the control group showed a clear and regular glomerular architecture and cell structure of the kidneys (Fig. 5a), while the model group mice exhibited a typical features of renal disease, such as arterial wall destruction, membrane proliferation and infiltration of inflammatory cells (Fig. 5b). In contrast, compared to the model group, the lesions in HLJDD treated mice were notably ameliorated with less damage to glomeruli and less inflammatory cells (Fig. 5c), which similar to prednisone group (Fig. 5e).

### The effect of HLJDD on the expression of renal *p*-STAT 3 protein in MRL/lpr mice

**Fig. 6:**
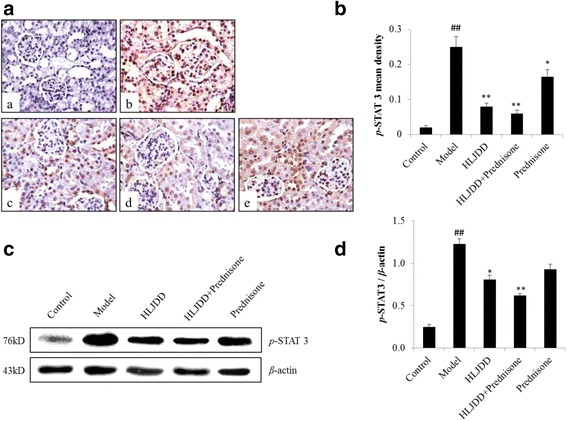
Effect of HLJDD on inhibiting phosphorylation of STAT 3 (*p*-STAT 3). **a** *p*-STAT 3 was measured on paraffin sections of the kidneys at the end of 8 week by immunohistochemistry (×400): (*a*) Control group; (*b*) Model group; (*c*) HLJDD group; (*d*) HLJDD + Prednisone group; (*e*) Prednisone group. **b** Mean density of *p*-STAT 3 was measured by Image-Pro Plus v 6.0. **c** Further analysis was to measure *p*-STAT 3 by Western blot. **d** Western blot of *p*-STAT 3 were subjected to semi-quantitative analysis by Image J. Values are presented as mean ± SD. ^##^ *p* < 0.01 versus control group, **p < 0.01 versus model group, *p < 0.05 versus model group. Western blot were repeated > 3 times

Previous studies in both humans and lupus prone mouse models have described that total levels of STAT 3 as well as the activation of STAT 3 are increased, compared to the healthy controls [48, 49]. Thus, the activation of *p*-STAT 3 were detected by the method of IHC and WB in the study. In IHC stained kidney sections, the expression of *p*-STAT 3 was observed mainly in the mesangial cells, glomerular endothelial cells and some renal tubular epithelial cells in MRL/lpr mice group (Fig. 6A). While consistent with the IHC results, the expression of *p*-STAT 3 protein was readily detected by WB in the model group (Fig. 6C). Conversely, *p*-STAT 3 expression was evidently decreased in mice treated with HLJDD (*p* < 0.01, *p* < 0.05, Fig. 6B, D), especially in the group of HLJDD combined prednisone (*p* < 0.01, Fig. 6B, D). Furthermore, IHC analysis also revealed that prednisone significantly restricted phosphorylation of STAT 3 compared to the model group (*p* < 0.05, Fig. 6B), though WB analysis showed no significant inhibition (Fig. 6D). These findings demonstrated that the expression of *p*-STAT 3 was blocked in the group of HLJDD-treated mice, which implying that STAT 3 may be one of the targets for HLJDD in the treatment of LN.

## Discussion

SLE is an autoimmune disease with the presence of immune complexes by autoantibodies, and these immune complexes would finally activating the complement pathway, forming kidney depositions and leading to the pathogenesis of LN [50]. Recently, although some immunosuppressive and anti-inflammatory agents such as steroid hormones and cyclosporine A are used in clinic patient for the prevention of SLE [51], there is no any common accepted therapy to reverse or prevent this process in humans, and the patients not only die from renal failure but also die from the opportunistic infection and cancer induced by the immunosuppression [43]. Thus, more and more attentions have been paid to the research and the development of effective therapy and agents for SLE diseases. Furthermore, the animal model of MRL/lpr mice is one of the commonly used spontaneous SLE models, and the mice symptoms are similar to human SLE, including immune complex-induced organ injury [52].

Chinese traditional herbal medicine is the natural therapeutic agent that used under the guidance of traditional Chinese medical theory, it has widely applied in alternative or complementary therapies and became more popular worldwide. Meanwhile, most herbal medicines are usually prescribed in combination in clinical application, in order to obtain the synergistic effects or to reduce the possible side effects, which are difficult to Western medicines [53]. HLJDD is a popular formula of TCM, which consist of 4 herbs. Previous chemical analysis show that the main active components in HLJDD including flavonoids, alkaloids and iridoids derived from its 4 herbs, and HLJDD played a much more clear anti-inflammatory effect as a whole prescription than that of its 4 typical herbs [17]. Moreover, recent researches also suggest that HLJDD exhibit immunity regulation, anti-complementary, anti-tumor, anti-hypoglycemic and anti-bacterial activities [54]. However, whether HLJDD alleviate the LN damage is not clear now. Thus, in the present study, we explored the reno-protective effects of HLJDD on LN MRL/lpr mice. As our knowledge, it is the first time to examine the effects of HLJDD on the therapy of SLE.

The lupus-like renal disease is a major feature in LN MRL/lpr mice [55], and the renal dysfunction could initially lead to mice death in the late stage of LN [56]. Proteinuria, a biomarker of the kidney inflammation, is an important cause for the mortality of MRL/lpr mice model, it is reflect the progression of renal disease. In the present study, HLJDD markedly increased the survival rate of MRL/lpr mice and suppressed the progression of proteinuria with more than 3-fold in the end of 8 weeks. Meanwhile, HLJDD dramatically decreased the spleen coefficient in MRL/lpr mice, which is one of the most important indexes in SLE progression. Furthermore, the kidney pathologic changes assessed by H&E staining demonstrated that HLJDD obviously alleviated the renal pathological injury, such as membrane proliferation, inflammatory cell infiltration and arterial wall destruction.

The key pathogenesis of LN is the continued increase in the autoantibodies productions and immune complex depositions in kidney [47]. The serum anti-dsDNA antibody levels is very specific for SLE [57], which is remain widely utilized to help establish the diagnosis of SLE and to predict nephritis activities [58]. While immune complex deposition can trigger a series of events that result in kidney inflammation and injury [59]. Our study showed that treatment with HLJDD remarkably reduced the serum levels of anti-dsDNA antibody and renal immune complex deposition complement C3 in MRL/lpr mice.

What’s more, an activated and dysregulated immune response is the characterize of SLE, and the pathophysiology of this regulatory defect is leaded by a distorted balance of regulatory cytokines. Thus, inflammation plays an indispensable role in the progression of LN [60, 61]. Recent reports showed that Th2 cytokines IL-6 and IL-10 have been shown to be important in the generation of SLE disease associated anti-dsDNA antibodies [62, 63]. In addition, Th1 cytokines IFN-γ, a potent immunomodulatory cytokine, is produced by T cell and natural killer cell (NK cell). It is a member of the interferon family that regulates the immune responses through activation of mononuclear macrophage, lymphocyte and NK cell, which is plays an important role in the pathogenesis of SLE [64]. The results in our study showed that oral administration of HLJDD could significantly inhibited the secretion of IL-6 and IL-10 in peripheral blood, which is consistent with the anti-dsDNA antibody levels. And the IFN-γ levels also remarkably decreased in HLJDD-treated group, compared to the model group.

STAT3, a transcription factor, is one of the member of JAK/STAT signaling pathway. It is activated downstream growth factors of a host, like the cytokines and chemokines [65]. The receptor of the STAT 3 will be dimerization and activation of Jak kinases, when its ligand bind to the receptor, which will in turn activate and phosphorylate STAT proteins. Then the phosphorylated STAT proteins will translocate to the nucleus and mediate gene transcription [66]. STAT 3 plays an important role in the development of cancer, chronic inflammation such as hepatitis, rheumatoid arthritis and etc. [67]. Several previous studies described that the activated STAT 3 in aglomerular mainly reside in diabetic nephropathy [68], kidney injury by HIV [69] and chronic proliferating immune complex glomerulonephritis [70]. In our study, compared to control group, the expression of *p*-STAT 3 was dramatically increased. While HLJDD-treated group significantly down-regulate the expression of *p*-STAT 3 through IHC and WB analysis. These results suggest that *p*-STAT 3 may be one of a promising therapeutic target for LN.

## Conclusion

Altogether, the study suggests that HLJDD has a potent reno-protective activity in the LN MRL/lpr mice. The treatment of HLJDD decreased the concentration of proteinuria, improves renal function and mitigates the renal pathology in LN. These effects followed by suppressing activation of *p*-STAT 3, restraining release of inflammatory cytokines, reducing autoimmune activity and by inhibiting macrophage infiltration in the kidney of LN MRL/lpr mice. Thus, HLJDD represents a potential therapy in preventing the LN progress, which could be a valuable alternative medicine for the effective treatment of SLE disease. Furthermore, the study also provided a proof of concept for the treatment of HLJDD in SLE disease.

## Abbreviations

anti-dsDNA: Anti-double stranded DNA
BUN: Blood urea nitrogen
CMC-Na: Carboxymethyl cellulose sodium salt
Cr: Creatinine
ECL: Enhanced chemi-luminescence system
ELISA: Enzyme-linked immunosorbent assay
H&E: Hematoxylin-eosin staining
HB: Huangbai
HL: Huanglian
HLJDD: Huang Lian Jie Du Decoction
HQ: Huangqin
IFN-γ: Interferon-gamma
IHC: Immunohistochemistry
IL-10: Interleukin-10
IL-6: Interleukin-6
LN: Lupus nephritis
NK cell: Natural killer cell
RT: Room temperature
SLE: Systemic lupus erythematosus
STAT3: Signal transducer and activator of transcription 3
TCM: Traditional Chinese medicine
WB: Western blot
ZZ: Zhizi
